# iGlarlixi for the simplification of more complex insulin regimens in type 2 diabetes patients with good glycaemic control

**DOI:** 10.3389/fendo.2026.1906632

**Published:** 2026-08-21

**Authors:** Zoltán J. Taybani, Balázs Bótyik, Dávid Major, Katalin Fehértemplomi, Agneta Veres, Máté Zatykó, Vince Fazekas-Pongor, István Panykó, Árpád Kézdi, Tamás Várkonyi, Ádám G. Tabák

**Affiliations:** 1 1^st^ Department of Endocrinology, Dr. Réthy Pál Member Hospital, Békés County Central Hospital, Békéscsaba, Hungary; 2 Institute of Preventive Medicine and Public Health, Semmelweis University Faculty of Medicine, Budapest, Hungary; 3 Health Sciences Division, Doctoral College, Semmelweis University, Budapest, Hungary; 4 Outpatient Clinic, Semmelweis University, Budapest, Hungary; 5 Department of Internal Medicine and Oncology, Semmelweis University Faculty of Medicine, Budapest, Hungary; 6 Rácz Károly Conservative Medicine Division, Doctoral College, Semmelweis University, Budapest, Hungary; 7 Department of Gynaecology and Obstetrics, Komárom-Esztergom County Szent Borbála Hospital, Tatabánya, Hungary; 8 Department of Internal Medicine, University of Szeged, Szeged, Hungary; 9 UCL Brain Sciences, University London, London, United Kingdom

**Keywords:** drug combinations, hypoglycemia, IGlarLixi, prospective studies, treatment simplification, type 2 diabetes, body mass index, glycated hemoglobin

## Abstract

**Aims:**

Knowledge on insulin treatment simplification with fixed ratio combinations (e.g. iGlarLixi, combination of insulin glargine and lixisenatide) is limited. Our aim was to examine long-term effectiveness and safety of treatment simplification with iGlarLixi in type 2 diabetes.

**Materials and methods:**

We report a single-centre, observational study of n=51 type 2 diabetes patients with HbA1c ≤ 7.5% (58 mmol/mol) who switched from basal-bolus or premixed insulins to iGlarLixi in 01/JAN/2017-30/JUN/2023 with up to 61-month follow-up. Outcomes were trajectories of HbA1c, BMI, insulin need, and risk of incident hypoglycaemia based on mixed linear models.

**Results:**

Participants (53% women) were 69.4 years old, BMI was 30.6 kg/m^2^, diabetes duration 13 years, HbA1c 6.4% [46 mmol/mol]), and insulin dose 0.46 IU/kg at baseline. HbA1c remained stable over follow-up. BMI dropped to 29.4 kg/m^2^ (slope: 0.54, 95%CI: 0.34-0.73 kg/month) in the first 7 months, then remained stable. Insulin dose dropped by 0.20 IU/kg (95%CI: 0.17-0.24) at baseline, then remained stable. Risk of hypoglycaemia decreased from 50 to 21% (odds ratio [OR] 0.88, 95%CI: 0.82-0.95/months) during the first 10 months, then remained stable. Subgroup analyses showed increasing HbA1c within the non-diabetic range in patients with very low and improvement in those with higher values. Furthermore, the subgroup with large BMI and low insulin dose at baseline had stable BMI trajectory (vs a decrease in all other subgroups).

**Conclusions:**

Simplification of complex regimens with iGlarLixi in patients with good glycaemic control maintains glycaemic control with weight loss, reduced risk of hypoglycaemia, and lower insulin dose over 42 months.

## Introduction

Basal-bolus (BB) insulin treatments provide effective glycaemic treatment for patients with type 2 diabetes. However, these treatments require a high degree of self-management ability and are associated with weight gain, risk of hypoglycaemia, and significant treatment burden (1).

Fixed-ratio combinations (FRCs) of a basal insulin (BI) and a glucagon-like peptide-1 receptor agonist (GLP-1RA) represent a novel approach to glycaemic treatment (2, 3). Examples of FRCs are the combination of insulin degludec and liraglutide [IDegLira] or insulin glargine and lixisenatide [iGlarlixi]. FRCs are administered once daily and have robust antihyperglycaemic effects given the complementary effect of their components (4). BIs primarily decrease fasting and preprandial glucose by limiting hepatic gluconeogenesis and increasing peripheral glucose uptake. GLP-1RAs inhibit glucagon production and enhance insulin secretion in a glucose-dependent way, thus ameliorating postprandial glucose excursions (2, 3, 5). FRCs applied as treatment intensification of oral antidiabetic therapy or BI-supported oral therapy have similar efficacy as BB or premixed insulin regimens (5–10).

A substantial proportion of patients are receiving unnecessarily complex insulin regimens that may be simplified without adverse effects (6). Simplification is recommended in cases of overtreatment when HbA1c is below the personalized target, or when HbA1c is at target but a simpler regimen could ensure the same glycaemia with less treatment burden. Furthermore, patients with HbA1c above target may also benefit from treatment simplification, especially if similar or better glycaemia can be reached (11, 12).

Common clinical opinion states that BB treatment is the ultimate insulin regimen with the highest glycaemic efficacy. However, there is increasing evidence that simplification from BB treatments to FRCs (mostly IDegLira) is possible without worsening glycaemic control in patients with both inadequate (HbA1c>7.5%) (13), or appropriate (HbA1c ≤ 7.5%) glycaemic control (14, 15). Furthermore, these simplified treatments require lower insulin doses and are associated with less hypoglycaemia, weight gain and treatment burden (13–15).

The current literature on insulin treatment simplification is limited in at least 3 aspects. First, none of the studies, except for Di Loreto et al. (16), have a follow-up >2 years and thus long-term effectiveness is debatable (13–15, 17–22). Second, studies either not reported separate data for iGlarLixi (13, 18), or only included IDegLira as the FRC in simplification studies (14–17, 19–22). Third, the only studies selecting people with adequate glycaemic control at baseline had up to 1-year follow-up and had IDegLira as the tested FRC (14, 15). Hence, we performed a retrospective cohort study to examine long-term effectiveness and safety of switching from complex insulin regimens to iGlarLixi in people with type 2 diabetes and an HbA1c ≤ 7.5% (58 mmol/mol).

## Materials and methods

### Study design and setting

This was a single-centre, retrospective, observational cohort study of type 2 diabetes patients regularly cared for diabetes at the Diabetes Centre of Dr. Réthy Pál Member Hospital, Békés County Central Hospital in Békéscsaba, Hungary. We included diabetes patients with relatively good glycaemic control who switched from basal-bolus or premixed insulin regimens to iGlarLixi between 01/JAN/2017 and 30/JUN/2023. Baseline characteristics and outcomes (glycaemic control, BMI, weight normalized insulin dose (normalized dose; total daily insulin dose [TDD]/body weight), and incident hypoglycaemia) were drawn from electronic health records (EHR, Medsol Smart healthcare system). All laboratory determinations were performed at the Central Laboratory of Dr. Réthy Pál Member Hospital, Békés County Central Hospital (see details in the **Supplementary Methods**).

Given the retrospective observational nature of the study, visits were scheduled as required for regular diabetes care. Individual participants had up to 11 visits including the baseline providing follow-up up to 61 months (median: 20, interquartile range [IQR: 10-36] months).

All study related procedures were performed in accordance with the ethical standards laid down in the 1964 Declaration of Helsinki and its later amendments. Ethical approval was obtained from the local ethical review board of Békés County Central Hospital (registration number: 33/2025). The need for informed consent was waived in accordance with Hungarian laws.

### Participants

**Figure 1:**
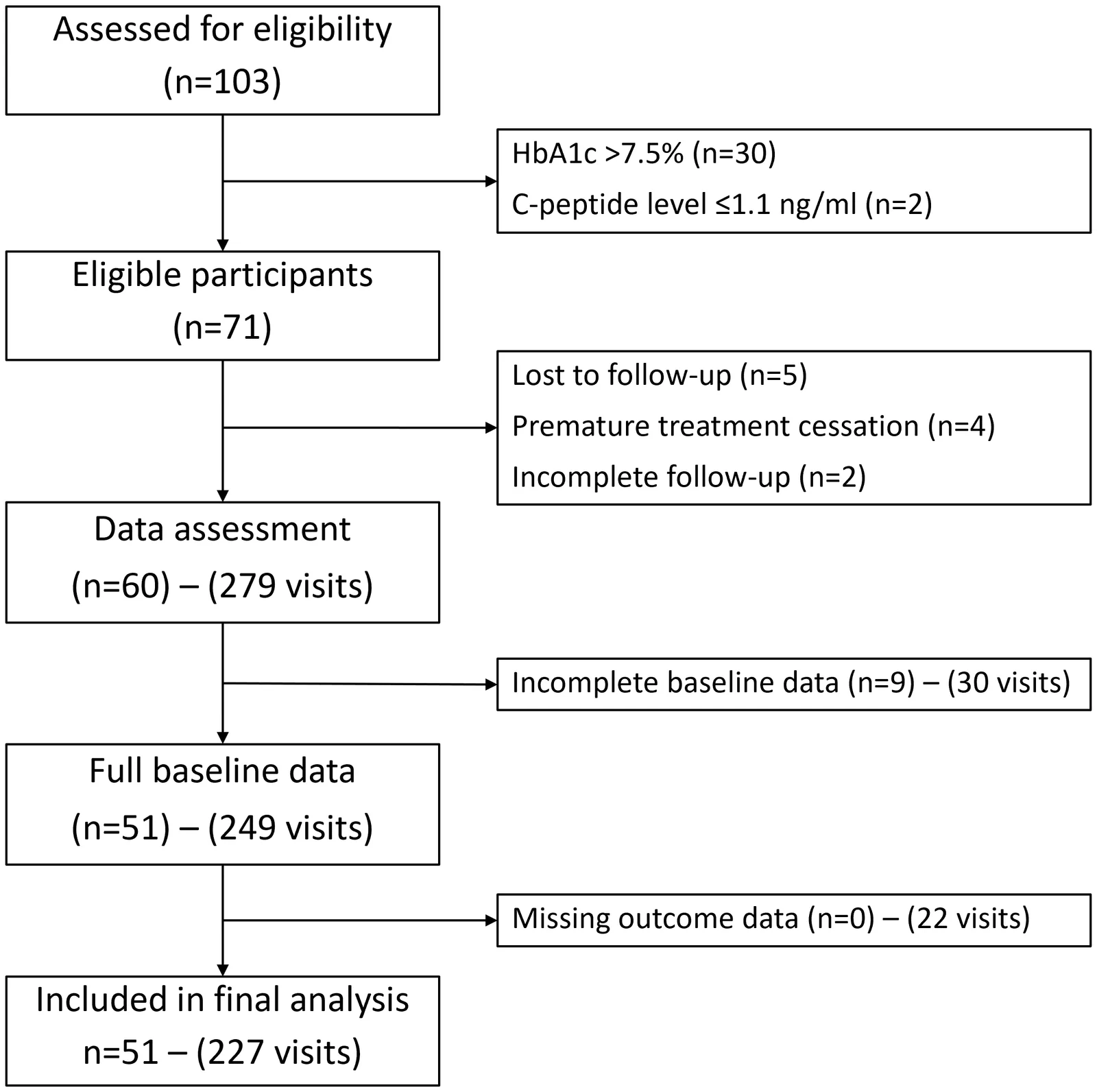
Study flow chart.

Altogether n=103 patients switched from a BB or premixed insulin to iGlarLixi. Details of the eligibility and exclusion criteria are described in the **Supplementary Table 1**. Of n=71 potentially eligible participants we excluded n=11 participants (lost to follow-up: n=5, premature treatment cessation before the first scheduled follow-up visit: n=4 (2 due to financial reasons, 2 due to perceived ineffectiveness), incomplete follow-up: n=2). Of the remaining 60 participants (84.5% of those eligible, n=279 measurement points), we further excluded n=9 participants (n=30 measurement points) due to incomplete baseline data and n=22 measurement points due to missing follow-up data leading to a final analytical sample of n=51 participants (71.8% of those eligible, n=227 measurement points) (**Figure 1**).

### Intervention

During the baseline visit, the starting dose of iGlarLixi was determined based on the estimated basal insulin requirement of the patient as described in the **Supplementary Methods**. The actual dose was determined on the treating physician’s discretion. On the day of iGlarLixi treatment initiation, all previously used insulins were discontinued. All patients received instructions on the use of the new drug delivery device but no extra educational session was performed on diabetes self-management.

Patients were instructed to self-titrate iGlarLixi based on the mean of pre-breakfast self-measured blood glucose for the last 3 days every three days. Patients were asked to modify the dose by 2 IUs if the mean glucose was outside the target range (5–6 mmol/L). Approximately 1–2 weeks after the baseline visit, a titration visit was scheduled with the diabetes educator to improve titration practices and screen for side effects.

We collected data on the use of other antidiabetic medications at each follow-up visit.

### Outcomes

The primary outcome was the trajectory of HbA1c over 42 months of follow-up. Secondary outcomes were trajectories of the proportion of participants with HbA1c <7.0%, BMI, normalized dose, and the absolute risk of incident hypoglycaemia (that is the percentage of patients experiencing ≥1 episode) in the preceding month.

At each clinic visit, the diabetes educator checked the patients’ physical or electronic diaries containing self-monitored blood glucose values for hypoglycaemic events (defined as any glucose level <3.9 mmol/l) for the last month and recorded the number of events (23). Furthermore, the treating physician also scanned the patients’ medical history for hospitalizations or emergency department visits for hypoglycaemia associated events in the National eHealth Infrastructure from the previous clinic visit. All events requiring professional help are recorded in the EHR with their exact dates. Hypoglycaemic events were defined as any of the above events within a month before the given visit.

Given, that we expected different shapes of these outcome trajectories in participants with lower or higher baseline values, we pre-specified the following subgroups using the median or the closest clinically interpretable conventional threshold: HbA1c analysis –<6.5% vs ≥6.5%; BMI analysis –<30 vs ≥30 kg/m^2^; normalized dose analysis – <0.44 vs ≥0.44 IU/day/kg. Furthermore, the selected HbA1c cutoff is in line with the recommendation of the American College of Physicians that suggests considering deintensification of pharmacological therapy if HbA1c is <6.5% (24).

Given that the weight lowering effect of GLP-1RAs is positively associated with its dose and the dose of lixisenatide parallels that of normalized dose, in addition to BMI subgroups, we further stratified our BMI analysis by normalized dose (<0.44 vs ≥0.44 IU/day/kg) (25).

### Statistical methods

Baseline characteristics are presented for all participants as well as stratified by baseline HbA1c (<6.5% vs ≥6.5%). Descriptive data are reported as mean ± standard deviation (SD) for continuous variables and counts (%) for categorical variables. Between group comparisons were done by independent samples t-tests for continuous and χ^2^-tests for categorical variables.

Given that each participant provided multiple observations over time, outcome trajectories were modelled by mixed linear models (for continuous outcomes) and generalized estimating equations (categorical outcome) with time, the pre-specified subgroups, and their interactions as predictors.

First, we modelled each outcome as a restricted cubic spline (RCS) of time with 5 knots (data points at 0, 5, 10, 17, and 29 months). This approach allows for a flexible non-linear effect of time on the outcome and provides a smooth graphical representation of the trajectory.

Next, after visual inspection of the graphs and the estimated marginal means (EMM) for bends and local minimum and maximum values over follow-up, we modelled the outcomes as linear splines of time. These models were further simplified to reach the most parsimonious parameterization. The fit of these models were acceptable based on the comparison of the log likelihood and information criteria (Akaike and Bayesean) values of the RCS and the linear spline models. These models allow the description and comparison of the rate of change for a given outcome over a pre-specified period over the follow-up time.

Finally, we calculated EMMs with 95% CIs based on the RCS models up to 42 months. We restricted visualization to 42 months, as the number of available measurements decreased substantially after month 42, resulting in increasingly sparse data and less reliable graphical estimates at later time points.

Given that the initiation of any new antidiabetic medication (in addition to iGlarLixi) may bias the outcome trajectories, we run a set of sensitivity analyses restricted to participants without incident antidiabetic medication use throughout the study.

Statistical analyses were done in StataNow 18.5 (StataCorp LLC, College Station, TX, US) and IBM SPSS Statistics for Windows 29.0.1.0 (IBM Corp, Armonk, NY, US). Statistical significance was set at p<0.05.

## Results

### Baseline characteristics

Of the 51 included Caucasian participants 27 (53%) were women. Participants were in average 69.4 years old, had a mean body weight of 87 kg corresponding to a BMI of 30.6 kg/m^2^ (45.1% had obesity and 45.1% had overweight). Mean duration of diabetes was ~13 years with an overall good glycaemic control (mean HbA1c: 6.4%). Almost 70% of the patients used metformin, 22% a sodium-glucose cotranspoter-2 inhibitor (SGLT2i). Most patients (88%) were on human insulin before the switch, 77% used 4 injections a day. TDD on the previous regimen was 39.4 IU corresponding to a normalized dose of 0.46 IU/kg. The starting dose of iGlarLixi was approximately half of the previous TDD and most patients started on the formulation with the higher lixisenatide concentration. Patients with higher and lower HbA1c showed similar characteristics except for an ~6 years longer diabetes duration and 1.2% higher HbA1c in the higher HbA1c group (**Table 1**).

**Table 1:** Baseline characteristics stratified by HbA1c.

Variable	Total	HbA1c subgroups		p-value
		<6.5% (48 mmol/mol)	≥6.5% (48 mmol/mol)	
*N*	51	27 (52.9%)	24 (47.1%)	
*Age, years*	69.4 ± 10.1	68.1 ± 11.5	70.9 ± 8.1	0.326
*Male, n (%)*	24 (47.1%)	14 (51.9%)	10 (41.7%)	0.467
*Weight, kg*	87.0 ± 17.4	88.6 ± 18.4	85.2 ± 16.5	0.490
*Height, cm*	168.3 ± 9.2	170.3 ± 9.0	166.0 ± 9.1	0.095
*BMI, kg/m^2^*	30.6 ± 5.4	30.5 ± 6.1	30.8 ± 4.7	0.877
*Diabetes duration, years*	12.6 ± 10.5	9.6 ± 10.6	16.0 ± 9.5	0.029
*Duration of insulin treatment, years*	6.2 ± 5.4	5.5 ± 5.7	7.1 ± 5.1	0.301
*HbA1c, %*	6.4 ± 0.7	5.9 ± 0.4	7.1 ± 0.3	<0.001
*HbA1c, mmol/mol*	46 ± 8	41 ± 4	54 ± 3	
*Fasting plasma glucose, mmol/L*	7.4 ± 2.4	6.8 ± 2.4	8.0 ± 2.2	0.075
*eGFR, mL/min/1.73 m^2^*	74.6 ± 15.6	73.9 ± 16.1	75.3 ± 15.3	0.743
*C-peptide, ng/mL*	2.97 ± 1.47	3.19 ± 1.59	2.72 ± 1.31	0.255
Previous antihyperglycaemic medications				
*Metformin, n (%)*	35 (68.6%)	20 (74.1%)	15 (62.5%)	0.374
*SGLT-2 inhibitor, n (%)*	11 (21.6%)	6 (22.2%)	5 (20.8%)	0.904
*Human insulin, n (%)*	45 (88.2%)	26 (96.3%)	19 (79.2%)	0.058
*Insulin injections*				0.98
*2 per day, n (%)*	10 (19.6%)	4 (14.8%)	6 (25.0%)	
*3 per day, n (%)*	2 (3.9%)	1 (3.7%)	1 (4.2%)	
*4 per day, n (%)*	39 (76.5%)	22 (81.5%)	17 (70.8%)	
*Weight normalized insulin dose, IU/kg*	0.46 ± 0.16	0.44 ± 0.17	0.48 ± 0.16	0.395
iGlarLixi				
*Starting dose*	17.8 ± 4.8	17.1 ± 5.0	18.7 ± 4.4	0.248
*iGlarLixi formulation*				0.90
*100IU/mL+50µg/mL*	47 (92.2%)	25 (92.6%)	22 (91.7%)	
*100IU/mL+33µg/mL*	4 (7.8%)	2 (7.4%)	2 (8.3%)	

Data are mean ± standard deviation or n (%).

P-values are given for 2-sample t-tests or χ2-tests as appropriate for the comparison between the HbA1c subgroups.

iGlarLixi is available in two different fixed-ratio combinations in Hungary (100/50, 100/33).

100IU/mL+33µg/mL: 100 IU/mL insulin glargine + 33µg/mL lixisenatide, 100IU/mL+50µg/mL: 100 IU/mL insulin glargine + 50µg/mL lixisenatide.

BMI, body mass index; eGFR, estimated glomerular filtration rate; iGlarLixi, insulin glargine/lixisenatide; SGLT-2, sodium-glucose cotransporter-2.

### Trajectory analysis

**Figure 2:**
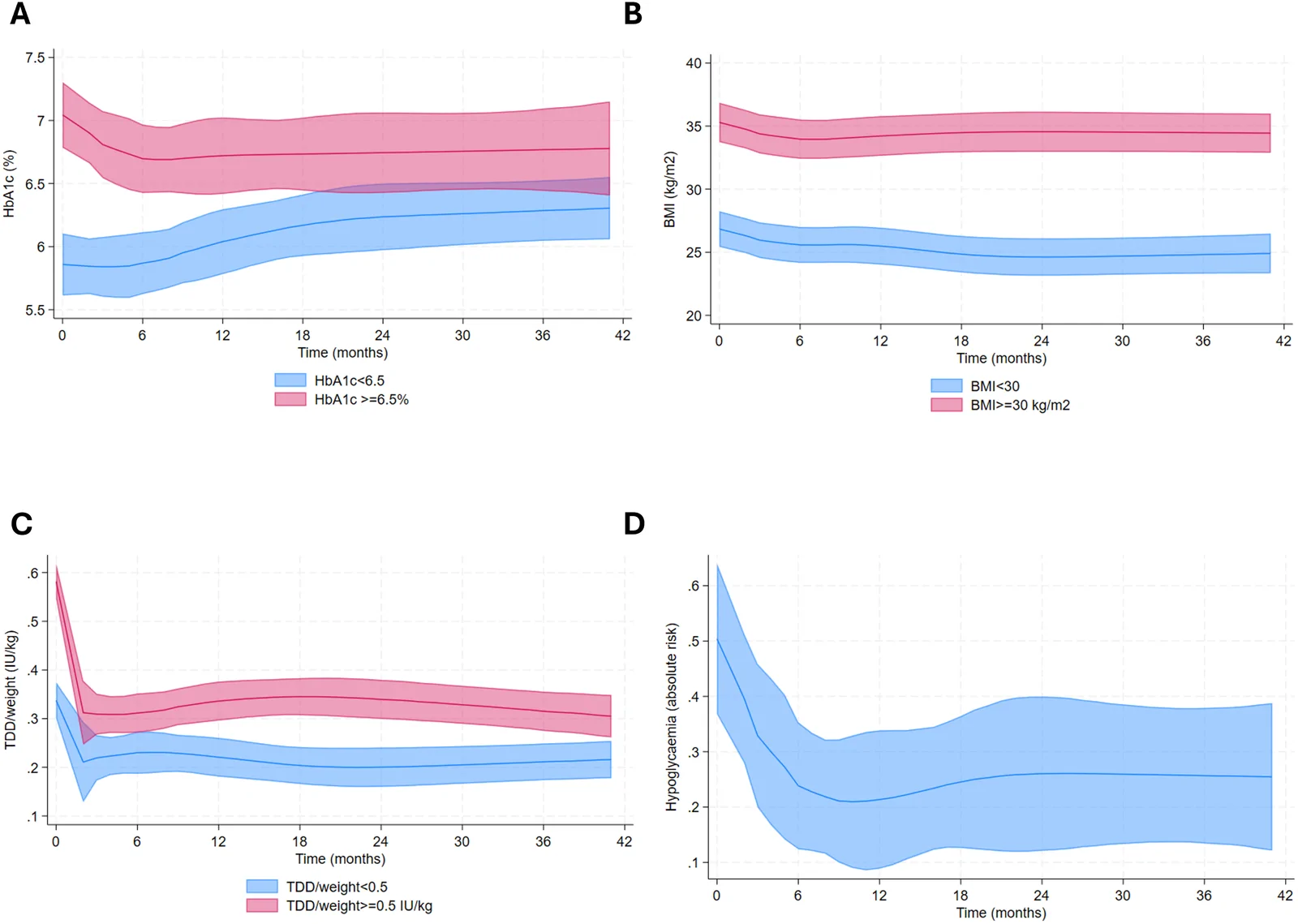
Estimated marginal means with 95% confidence bands of HbA1c **(A)**, BMI **(B)**, normalized dose by subgroups **(C)**, and absolute risk of hypoglycaemia in the preceding month for the whole population **(D)** based on mixed model analyses using restricted cubic splines. To convert HbA1c (%) to mmol/mol, the conversion formula is: HbA1c (mmol/mol)=10.9xHbA1c(%)-23.5.

**Figure 3:**
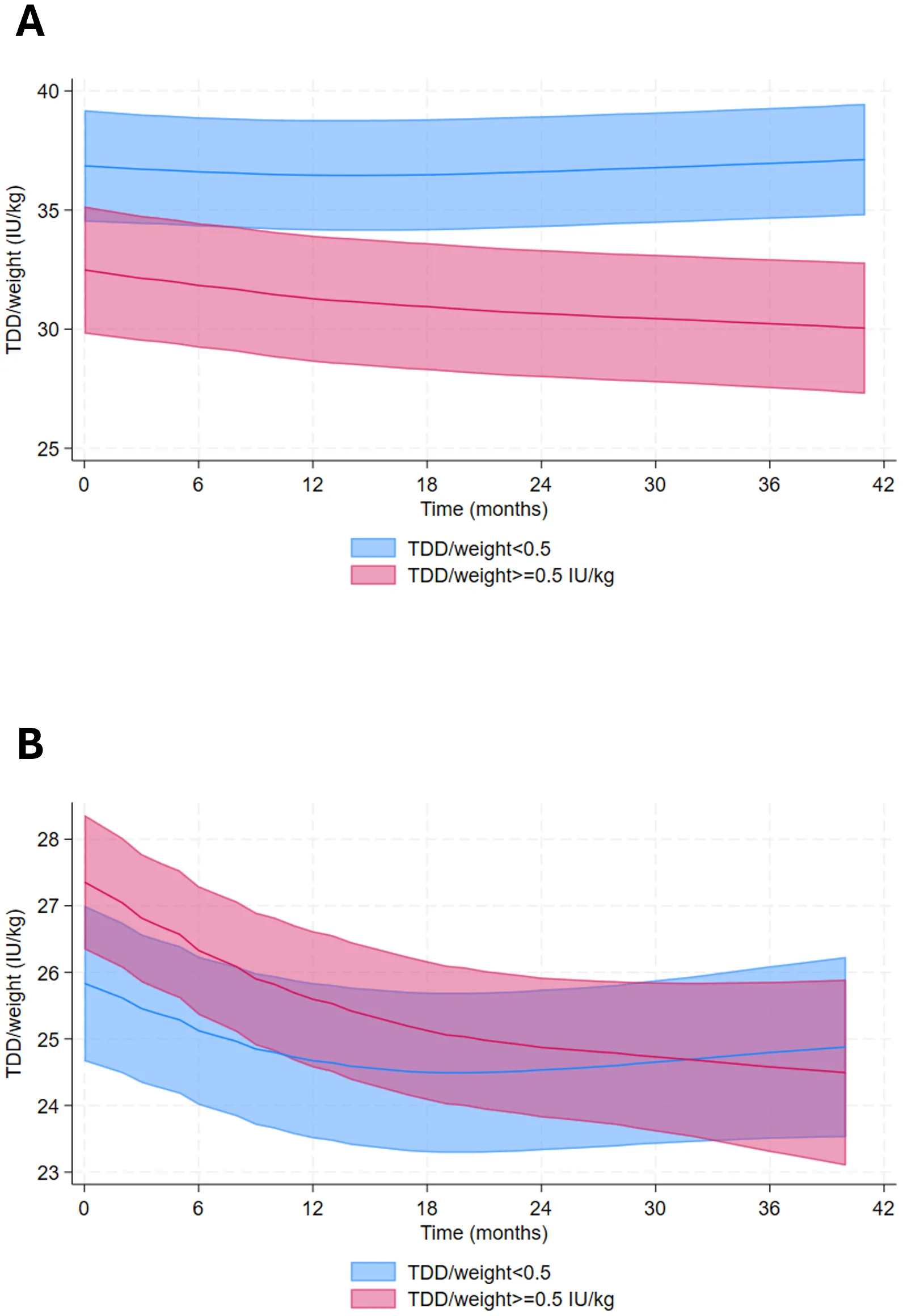
Estimated marginal means with 95% confidence bands of BMI by baseline weight normalized insulin dose among participants with higher (≥30 kg/m^2^ – **A**) and lower BMI (<30 kgm^2^– **B**) at baseline based on mixed model analyses using restricted cubic splines.

The included 51 patients provided altogether 227 data points (median 5, [IQR] 3–7 measures/person). Median follow-up was 20 (IQR: 10-36) months (**Table 2**, **Figures 2**, **3**).

**Table 2:** Estimated values (and 95% confidence intervals) of HbA1c, body weight, body mass index (BMI), total daily insulin dose, weight normalised insulin dose, and absolute risk of hypoglycaemia in the preceding month based on mixed model analyses using restricted cubic splines.

Variable	Baseline	6 months	12 months	24 months	36 months	42 months
	EMM (95%CI)	EMM (95%CI)	EMM (95%CI)	EMM (95%CI)	EMM (95%CI)	EMM (95%CI)
HbA1c, %						
*Overall*	6.4 (6.2 to 6.6)	6.3 (6.0 to 6.5)	6.4 (6.1 to 6.6)	6.5 (6.2 to 6.7)	6.5 (6.3 to 6.8)	6.6 (6.3 to 6.8)
*Proportion <7%, %*	68.6 (55.9 to 81.3)	68.8 (56.0 to 81.6)	68.8 (55.9 to 81.8)	68.8 (55.6 to 81.9)	69.0 (56.0 to 81.9)	69.1 (56.0 to 82.1)
Stratified by subgroups						
*<6.5%*	5.9 (5.6 to 6.1)	5.9 (5.6 to 6.1)	6.0 (5.8 to 6.3)	6.2 (6.0 to 6.5)	6.3 (6.0 to 6.5)	6.3 (6.0 to 6.6)
*≥6.5%*	7.0 (6.8 to 7.3)	6.7 (6.4 to 7.0)	6.7 (6.4 to 7.0)	6.7 (6.4 to 7.1)	6.8 (6.4 to 7.1)	6.8 (6.4 to 7.2)
*Difference*	**1.2 (0.8 to 1.5)**	**0.8 (0.5 to 1.2)**	**0.7 (0.3 to 1.1)**	**0.5 (0.1 to 0.9)**	**0.5 (0.1 to 0.9)**	**0.5 (0.1 to 1.0)**
Weight, kg						
*Overall*	87.0 (82.0 to 92.0)	83.3 (78.3 to 88.3)	83.5 (78.5 to 88.6)	82.9 (77.8 to 88.0)	83.1 (78.1 to 88.1)	83.2 (78.1 to 88.3)
BMI, kg/m^2^						
*Overall*	30.7 (29.1 to 32.2)	29.3 (27.8 to 30.9)	29.5 (27.9 to 31.1)	29.2 (27.6 to 30.8)	29.3 (27.7 to 30.9)	29.4 (27.7 to 31.0)
Stratified by subgroups						
*<30 kg/m2*	26.8 (25.4 to 28.2)	25.6 (24.2 to 27.0)	25.5 (24.0 to 26.9)	24.6 (23.1 to 26.1)	24.8 (23.3 to 26.3)	24.9 (23.3 to 26.5)
*≥30 kg/m2*	35.3 (33.7 to 36.8)	34.0 (32.4 to 35.5)	34.2 (32.6 to 35.9)	34.6 (33.0 to 36.1)	34.5 (32.9 to 36.0)	34.4 (32.9 to 36.0)
*Difference*	**8.5 (6.4 to 10.5)**	**8.4 (6.3 to 10.5)**	**8.7 (6.6 to 10.9)**	**9.9 (7.8 to 12.1)**	**9.7 (7.5 to 11.8)**	**9.5 (7.3 to 11.7)**
Total daily insulin dose, IU						
*Overall*	39.0 (36.1 to 41.9)	19.6 (16.7 to 22.6)	24.0 (20.9 to 27.1)	21.8 (18.6 to 25.0)	22.0 (19.0 to 25.0)	22.2 (19.1 to 25.3)
Weight normalized insulin dose, IU/kg						
*Overall*	0.46 (0.42 to 0.49)	0.24 (0.21 to 0.28)	0.29 (0.26 to 0.33)	0.27 (0.23 to 0.31)	0.27 (0.23 to 0.31)	0.27 (0.24 to 0.31)
Stratified by subgroups						
*<0.44 IU/kg*	0.33 (0.30 to 0.37)	0.23 (0.19 to 0.27)	0.22 (0.18 to 0.26)	0.20 (0.16 to 0.24)	0.21 (0.17 to 0.25)	0.22 (0.18 to 0.25)
*≥0.44 IU/kg*	0.58 (0.55 to 0.62)	0.31 (0.27 to 0.35)	0.34 (0.30 to 0.38)	0.34 (0.30 to 0.38)	0.32 (0.27 to 0.36)	0.30 (0.26 to 0.35)
*Difference*	**0.24 (0.19 to 0.30)**	**0.08 (0.02 to 0.14)**	**0.11 (0.06 to 0.17)**	**0.13 (0.08 to 0.20)**	**0.10 (0.05 to 0.16)**	**0.09 (0.03 to 0.15)**
Incident hypoglycaemia in the preceding month, %						
*Total*	50.4 (36.8 to 64.0)	23.9 (12.4 to 35.3)	21.4 (8.9 to 33.9)	26.0 (12.1 to 40.0)	25.7 (13.4 to 37.9)	25.5 (12.1 to 38.8)

CI, confidence interval; EMM, estimated marginal mean.

To convert HbA1c (%) to mmol/mol, the conversion formula is: HbA1c (mmol/mol)=10.9xHbA1c(%)-23.5.

Statistically significant subgroup differences are marked with bold numbers.

#### Glycaemic trajectories

Overall glycaemia measured as the proportion of patients with HbA1c<7% did not change over follow-up. Mean HbA1c decreased non-significantly from 6.4% to 6.3% (slope: -0.03, 95%CI: -0.06–0.01%/month) in the first 6 months. It was followed by a non-significant increase (slope: 0.02, 95%CI: -0.00–0.04%/month) to 6.5% for 12 months, then it remained stable (**Table 2**).

Subgroups of patients with higher and lower baseline HbA1c (<6.5% vs ≥6.5%) revealed different patterns. People with lower baseline HbA1c showed no change in the first 8 months, but their HbA1c values started to increase thereafter (slope: 0.010, 95%CI: 0.003–0.016%/month) but remained below 7%. In contrast, individuals with higher baseline HbA1c experienced a drop in the first eight months (slope: 0.04, 95%CI: 0.01–0.08%/month), then HbA1c remained stable (**Table 2**, **Figure 2A**).

#### Body weight and BMI trajectories

The mean body weight dropped from 87.0 to 83.2 kg (slope: 0.54, 95%CI: 0.34–0.73 kg/month) in the first 7 months, then it remained stable. BMI followed a similar trajectory with a drop from 30.7 to 29.4 kg/m^2^ (slope: 0.19, 95%CI: 0.12–0.26 kg/m^2^/month) in the first 7 months, then it remained stable (**Table 2**).

Subgroups based on BMI (<30 vs ≥30 kg/m^2^) showed more nuanced patterns. BMI decreased similarly in both BMI subgroups in the first 7 months (slope for BMI<30: 0.17, 95%CI: 0.07–0.28 kg/m^2^/month; slope for BMI≥30: 0.19, 95%CI: 0.09–0.30 kg/m^2^/month). However, BMI in the lower BMI subgroup decreased further (slope: 0.06, 95%CI: 0.002–0.12 kg/m^2^/month) for another 17 months before stabilizing, while the BMI in the higher BMI subgroup did not change after the first 7 months (**Table 2**, **Figure 2B**).

#### Trajectories of TDD and normalized dose

Mean TDD dropped from 39.4 IU by 18.0 IU (95%CI: 15.0–21.0) due to the simplification of therapy at baseline without any changes thereafter. Normalized dose (TDD/weight) followed a similar course with a drop from 0.46 IU/kg by 0.20 IU/kg (95%CI: 0.17–0.24) at baseline, followed by no changes (**Table 2**).

Again, participants stratified by normalized dose showed important differences. The immediate drop in normalized dose (due to change of medication) was approximately twice as large in the group with higher vs lower normalized dose at baseline (0.29, 95%CI: 0.22–0.35 vs 0.13, 95%CI: 0.06–0.20 IU/kg). The group with the lower normalized dose showed no further changes thereafter. In contrast, the group with higher normalized dose had an increase (slope: 0.0026, 95%CI: 0.0004–0.0048 IU/kg/month) until 20 months, followed by a similar decline (slope: 0.0022, 95% CI: 0.0039–0.0005 IU/kg/month) (**Table 2**, **Figure 2C**).

#### Trajectories of hypoglycaemia risk

Altogether 2 severe hypoglycaemia events were reported by 2 patients during the whole follow-up. Both patients recovered without complications after treatment.

The risk of hypoglycaemia in the preceding month decreased substantially from 50 to 21% (odds ratio [OR] 0.88, 95%CI: 0.82–0.95/months) during the first 10 months after treatment simplification and remained relatively unchanged thereafter (**Table 2**, **Figure 2D**).

#### Prespecified analysis of BMI trajectories stratified by normalized dose

Further stratification of the BMI trajectories provided evidence that the subgroups with lower BMI (<30 kg/m^2^) followed similar decreasing patterns in the first 19 months of follow-up and stabilized thereafter irrespective of baseline normalized dose (<0.44 vs ≥0.44 IU/day/kg) with overlapping confidence intervals (**Figure 3A**).

In contrast, those patients with obesity at baseline followed different trajectories based on their insulin need. The BMI trajectory of those patients with a lower normalized dose was completely flat, while those with a higher normalized dose showed a decreasing trajectory until 14 months of follow-up (slope: 0.09, 95%CI: 0.01–0.17 kg/m^2^) with no changes thereafter (**Figure 3B**).

### Sensitivity analyses

The sensitivity analysis restricted to n=36 participants without the initiation of any new antidiabetic medication throughout the study largely confirmed the results of the main analyses with similar shapes of overall and subgroup trajectories (**Supplementary Table 2**, **Supplementary Figures 1**, **2**).

## Discussion

### Short summary

Our study provides strong observational evidence that after the simplification of more complex insulin regimens to iGlarlixi, overall glycaemic control remains stable, BMI decreases by 1.3 kg/m^2^ and normalized dose, as well as risk of hypoglycaemia drops to less than half of the baseline values in a cohort of type 2 diabetes patients with good glycaemic control at baseline for at least 42 months.

Furthermore, our subgroup analyses provide evidence for important differences between trajectories based on baseline characteristics. First, patients with very low baseline HbA1c showed a shallow increase in glycaemia within the non-diabetic range, while those with higher HbA1c at baseline showed improving glycaemia. Second, while patients with both lower and higher BMI showed improving BMI, the decrease lasted longer and resulted in a larger drop among those with a lower baseline value. Third, patients with larger normalized dose at baseline showed a larger drop in their normalized dose, however they required further titration, while the lower normalized dose group required only non-significant dose modifications. Lastly, we found that compared with improving BMI trajectories in all other subgroups, the subgroup with a large BMI and a low normalized dose at baseline showed a stable BMI trajectory.

### Importance of the findings

Traditionally, multiple daily insulin injection therapy was thought to be the most effective glucose-lowering regimen given its ability to lower glucose levels in a dose-dependent manner without a dose maximum. However, its real-world effectiveness is limited by its burdensome use and increased risk of hypoglycaemia (26). Indeed, insulin omission or non-adherence is frequent (27, 28), and is often associated with inadequate glucose control and higher healthcare costs (29–31). Furthermore, the majority of individuals feel that insulin regimens are restrictive and think that it is hard to live a normal life while managing diabetes with insulin. It is not surprising thus that patients would prefer to use fewer daily injections and want a more flexible treatment (27).

Thus, FRCs applied instead of complex insulin regimens represent an attractive treatment option as FRCs are administered once daily and are easier to titrate. This theoretical benefit is clearly shown by the improved scores on the Diabetes Treatment Satisfaction Questionnaire (DTSQ) in deintensification studies (18, 19). In our study, the number of daily insulin injections dropped from a median of 4 to 1 after switching to iGlarLixi, which translates to ~1000 fewer injections/year compared to the previous regimen.

A potential indication for deintensification of complex insulin regimens to FRCs is overtreatment. Overtreatment of type 2 diabetes is characterized by lower HbA1c and/or higher risk of hypoglycaemia compared to personalized goals (26). Overtreatment with hypoglycaemic agents is a common but a generally unrecognized problem in type 2 diabetes (32–34). In addition, overtreatment should be considered when well-controlled patients use an unnecessarily complex regimen instead of simpler options that would provide similar glycaemia with a lower treatment burden (14, 35). Our trial recruited participants with both types of overtreatments. Approximately half of our participants had an HbA1c below 6.5% representing the form of overtreatment defined by guidelines (26, 36), the other half had well-controlled diabetes but were using potentially unnecessarily complex regimens.

### Results in context of the literature

Comparison of our findings to the literature is hindered by the fact that most previous studies focused on the other commercially available FRC (IDegLira) (14–17, 19–22), reported short term outcomes (≤12 months) (13–15, 19–22), and included participants with poor glycaemic control (>8%) (2, 13, 16, 17, 19–22). In contrast to our observation of no significant change in HbA1c, all those studies with elevated baseline HbA1c reported significant HbA1c improvements in the range of 0.3-1.1% (3–12 mmol/mol) (2, 13, 16, 17, 19–22). It should be noted that despite decreasing glycaemic values during follow-up, HbA1c values remained mostly >7% (53 mmol/mol), meaning that less than half of the participants reached the generally recommended 7% (53 mmol/mol) threshold (2, 13, 16, 17, 19–22). The observed changes in other outcomes well correspond to our observation with BMI decreases in the range of 0.8–2.5 kg/m^2^, TDD decreases of 20–32 IU/day, and substantial decreases in the risk of hypoglycaemia (2, 13–17, 19–22).

An important, yet unanswered question is, whether FRCs can provide long-term glycaemic control after deintensification. In addition to our study, there are only 3 studies that have >1-year duration, but none of these investigated the effectiveness of solely iGlarLixi and all have baseline mean HbA1c values >8% (64 mmol/mol) (16–18). While all 3 studies report improved HbA1c compared to baseline, none examined glycaemic trajectories, thus are unable to determine changes in effectiveness over time. Furthermore, the BEYOND randomized trial that compared multiple daily injection therapy with the use of FRCs reported reduced efficacy of the FRCs compared to basal-bolus therapy (HbA1c change 0.7 vs 1.3%) in the intention-to-treat population (18). Again, the changes in BMI and the risk of hypoglycaemia were similar to that observed by us, however our is the only study reporting sustained effects after early improvements in these outcomes.

There are 2 other reports that recruited well-controlled patients (from the same authors) but using a different setting: the FRC was different (IDegLira) and they reported short-term (≤1 year) outcomes (14, 15). Similarly to the current findings, no change in HbA1c was reported and the other outcomes showed similar improvements.

Overall, our findings extend current evidence by reporting similar effectiveness and safety of iGlarLixi to IDegLira and by reporting sustained effectiveness up to 42 months.

Our subgroup analyses significantly increase our knowledge on the effectiveness of these medications. We found improving glycaemia even among those with only slightly elevated HbA1c (6.5–7.5%). We observed larger and longer effects on BMI among patients with lower BMI (<30 kg/m^2^) and among patients with higher BMI who required higher insulin doses (>0.44 IU/kg) at baseline. We also found that further dose titration is required in those with larger baseline doses. The finding of no decrease in HbA1c among patients with lower HbA1c at baseline is expected and desirable. Our findings on changes in BMI probably reflect the fact that the BMI lowering effect of GLP-1RAs is dose dependent and GLP-1RA dose changes parallel with insulin dose in FRCs (25). In line with this reasoning, we suggest the use of free combination of a GLP-1RA and basal insulin in patients with obesity with relatively low insulin doses, as this allows the use of maximally tolerable dose of GLP-1RA.

### Strengths and limitations

Ours is one of the longest studies on the effectiveness of any FRCs and the first in which iGlarLixi was used for insulin treatment simplification. We were enrolling patients with relatively well-controlled diabetes. Our clinical experience and previous trials with IDegLira suggested that the best candidates for insulin treatment simplification with FRCs could be those with an HbA1c<7.5%, a positive C-peptide, and a relatively low insulin dose (14, 15). Given the long follow-up and the large number of visits, the relatively moderate number of participants provided ample statistical power even for subgroups analyses. Although our study was retrospective, both data collection, treatment initiation, and intensification followed standardized protocols that enhance interpretation of the findings. Our statistical analysis optimized the use of repeated data using state-of-the-art methods. The converging results from our main analysis and the sensitivity analysis further supports our main conclusions.

Our study also has limitations that must be acknowledged. First, we had no control group and thus our findings are prone to overestimation of treatment benefits, although no education (except for the injection device and dose titration) was provided to the participants. Given the single centre design and the relatively small number of participants of our study, external validity (especially for the subgroup analyses) of the findings is limited and require further confirmation in large independent samples. As with any retrospective data collection, our analysis is prone to different types of bias. While this is unlikely to influence laboratory and objectively measured outcomes (such as HbA1c and BMI), both insulin dose and hypoglycaemia were self-reported. We believe that our standardized data collection minimized selective under- or overreporting of hypoglycaemic events. We used a non-fasting C-peptide cutoff of >1.1 ng/mL as an inclusion criterion to exclude type 1 diabetes, latent autoimmune diabetes, or type 2 diabetes with severe insulin deficiency. It could be argued that this cut-off value is too strict and a more relaxed inclusion criteria could increase the eligibility without loss of effectiveness (37).

### Conclusion

We have shown that simplification of premixed or basal-bolus insulin regimens with iGlarlixi in patients with baseline HbA1c<7.5% and a positive C-peptide can improve or maintain glycaemic control provided by previous regimens with additional benefits such as weight loss, reduced risk of hypoglycaemia, lower weight normalized insulin dose, and fewer injections. In fact, our trajectory analyses clearly showed that these improvements were sustained over for at least 42 months. Our subgroup analyses explored important clinical characteristics that may help predicting changes in important outcomes after deintensification, as well as reducing clinical inertia limiting treatment simplification.

## Data Availability

The raw data supporting the conclusions of this article will be made available by the authors, without undue reservation.
