# Supplementary material for: iGlarlixi for the simplification of more complex insulin regimens in type 2 diabetes patients with good glycaemic control
Source: Front Endocrinol (Lausanne). 2026 Aug 21;17:1906632. doi: 10.3389/fendo.2026.1906632 (PMC13541476; doi:10.3389/fendo.2026.1906632)

iGlarlixi for the simplification of more complex insulin regimens in type 2 diabetes patients with good glycaemic control

Supplementary Material

Zoltán J. Taybani[1], Balázs Bótyik[1], Dávid Major[2,3], Katalin Fehértemplomi[1], Agneta Veres[1], Máté Zatykó[1], Vince Fazekas-Pongor[2,4], István Panykó[5,6,7], Árpád Kézdi[5,6], Tamás Várkonyi[8], Ádám G. Tabák[2,5,9]

[1] 1st Department of Endocrinology, Dr. Réthy Pál Member Hospital, Békés County Central Hospital, Békéscsaba, Hungary

[2] Institute of Preventive Medicine and Public Health, Semmelweis University Faculty of Medicine, Budapest, Hungary

[3] Health Sciences Division, Doctoral College, Semmelweis University, Budapest, Hungary

[4] Outpatient Clinic, Semmelweis University, Budapest, Hungary

[5] Department of Internal Medicine and Oncology, Semmelweis University Faculty of Medicine, Budapest, Hungary

[6] Rácz Károly Conservative Medicine Division, Doctoral College, Semmelweis University, Budapest, Hungary

[7] Department of Gynaecology and Obstetrics, Komárom-Esztergom County Szent Borbála Hospital, Tatabánya, Hungary

[8] Department of Internal Medicine, University of Szeged, Szeged, Hungary

[9] UCL Brain Sciences, University London, London, United Kingdom

Supplementary Materials and Methods

Study related data collection

All laboratory determinations were performed at the Central Laboratory of Dr. Réthy Pál Member Hospital, Békés County Central Hospital. Serum creatinine and C-peptide (Elecsys C-Peptide reagent, Roche Magyarország, Budapest, Hungary) were measured on a Roche Cobas 6000 analyser (Roche Magyarország, Budapest, Hungary) at baseline. Estimated glomerular filtration rate (eGFR) was calculated using the CKD-EPI equation.[1] Fasting plasma glucose was determined after at least 8 hours of fasting using a glucose oxidase method at each visit. HbA1c was measured with a Bio-Rad Variant II automated HbA1c analyser using Variant II Turbo HbA1c Kit-2.0 (Bio-Rad Magyarország Kft., Budapest, Hungary) at each visit.

Determination of the starting dose of iGlarLixi

During the baseline visit, the starting dose of iGlarLixi (combination of insulin glargine and lixisenatide) was determined based on the estimated basal insulin requirement of the patient: the estimated dose was the dose of the basal analogue if a basal analogue insulin was used, and half of the total daily insulin dose (TDD) if a human basal insulin was used. If the estimated dose was ≤30 IU, iGlarLixi 100/50 (100 IU/mL insulin glargine + 50 μg/mL lixisenatide) was started at a maximum of 20 dose steps. If the estimated dose was ≥30 IU, iGlarLixi 100/30 was started at a maximum of 30 dose steps.

Reference

1. Levey AS, Stevens LA, Schmid CH, et al. A new equation to estimate glomerular filtration rate. *Ann Intern Med*. May 5 2009;150(9):604-12. doi:10.7326/0003-4819-150-9-200905050-00006

Supplementary Table S1 – Eligibility and exclusion criteria

**Eligibility criteria**	**Exclusion criteria**
• ≥18 years old<br>• on BB or premixed (≥2 injection/day) insulin regimens for ≥3 months<br>• HbA1c ≤7.5% at baseline<br>• insulin need ≤0.8 IU/kg at baseline<br>• random, non-fasting serum C-peptide >1.1 ng/mL (normal range 1.1–4.1 ng/mL) at baseline	• the diagnosis of other (not type 2) diabetes<br>• treatment with a GLP-1RA or an anti-obesity drug within 3 months of initiation of iGlarLixi<br>• significant anaemia (haemoglobin <100 g/L)<br>• active cancer<br>• estimated glomerular filtration rate <30 mL/min/1.73 m^2
Abbreviations: BB: basal-bolus; GLP-1RA: glucagon-like peptide-1 receptor agonist; HbA1c: glycated haemoglobin; iGlarLixi: combination of insulin glargine and lixisenatide	

Supplementary Table S2 – Estimated values (and 95% confidence intervals) of HbA1c, body weight, body mass index (BMI), total daily insulin dose, weight normalised insulin dose, and absolute risk of hypoglycaemia in the preceding month based on mixed model analyses using restricted cubic splines in the subgroup of patients on stable non-injectable antidiabetic treatment

	**Baseline EMM (95%CI)**	**6 months EMM (95%CI)**	**12 months EMM (95%CI)**	**24 months EMM (95%CI)**	**36 months EMM (95%CI)**	**42 months EMM (95%CI)**
HbA1c, %						
Overall	6.5 (6.2 to 6.7)	6.3 (6.0 to 6.5)	6.2 (5.9 to 6.5)	6.3 (6.0 to 6.6)	6.5 (6.2 to 6.8)	6.5 (6.2 to 6.8)
Subgroups						
<6.5%	5.9 (5.6 to 6.2)	5.8 (5.5 to 6.1)	5.8 (5.4 to 6.1)	5.9 (5.6 to 6.2)	6.1 (5.8 to 6.4)	6.1 (8.8 to 6.5)
≥6.5%	7.0 (6.7 to 7.3)	6.7 (6.4 to 7.0)	6.7 (6.4 to 7.0)	6.7 (6.4 to 7.1)	6.8 (6.5 to 7.2)	6.9 (6.5 to 7.2)
Difference	**1.2 (0.7 to 1.6)**	**1.0 (0.6 to 1.4)**	**1.0 (0.5 to 1.4)**	**0.8 (0.3 to 1.3)**	**0.7 (0.3 to 1.2)**	**0.7 (0.2 to 1.2)**
BMI, kg/m²						
Overall	29.9 (28.2 to 31.6)	28.4 (26.7 to 30.1)	28.5 (26.8 to 30.3)	28.4 (26.7 to 30.2)	28.6 (26.9 to 30.3)	28.7 (27.0 to 30.4)
Subgroups						
<30 kg/m2	27.0 (25.6 to 28.4)	25.7 (24.2 to 27.1)	25.4 (23.9 to 27.0)	24.3 (22.7 to 25.8)	24.4 (22.2 to 26.6)	24.5 (22.8 to 27.2)
≥30 kg/m2	34.5 (32.7 to 36.3)	32.9 (31.1 to 34.7)	33.2 (31.4 to 35.1)	34.0 (32.2 to 35.8)	33.9 (32.1 to 35.7)	33.9 (32.1 to 35.7)
Difference	**7.5 (5.2 to 9.8)**	**7.3 (4.9 to 9.6)**	**7.8 (5.4 to 10.2)**	**9.7 (7.3 to 12.1)**	**9.5 (6.7 to 12.4)**	**9.4 (6.2 to 12.7)**
Weight normalized insulin dose, IU/kg						
Overall	0.50 (0.46 to 0.54)	0.28 (0.23 to 0.32)	0.29 (0.25 to 0.33)	0.28 (0.24 to 0.32)	0.27 (0.23 to 0.31)	0.27 (0.23 to 0.31)
Subgroups						
<0.44 IU/kg	0.35 (0.31 to 0.40)	0.22 (0.16 to 0.27)	0.21 (0.16 to 0.26)	0.19 (0.14 to 0.24)	0.20 (0.15 to 0.25)	0.21 (0.16 to 0.25)
≥0.44 IU/kg	0.59 (0.56 to 0.63)	0.32 (0.25 to 0.36)	0.34 (0.30 to 0.38)	0.33 (0.29 to 0.37)	0.30 (0.25 to 0.34)	0.29 (0.24 to 0.33)
Difference	**0.24 (0.18 to 0.30)**	**0.10 (0.03 to 0.17)**	**0.13 (0.06 to 0.19)**	**0.14 (0.07 to 0.20)**	**0.10 (0.03 to 0.16)**	**0.08 (0.01 to 0.15)**
Incident hypoglycaemia in the preceding month, %						
Total	54.7 (38.6 to 70.8)	23.1 (9.8 to 36.4)	19.4 (4.3 to 34.6)	22.0 (5.9 to 38.1)	11.8 (0 to 24.7)	9.3 (0 to 22.2)

CI: confidence interval, EMM: estimated marginal mean

To convert HbA1c (%) to mmol/mol, the conversion formula is: HbA1c (mmol/mol)=10.9xHbA1c(%)-23.5

Supplementary Figure S1 – Estimated marginal means with 95% confidence bands of HbA1c (Panel A), of BMI (Panel B), of normalized dose by subgroups (Panel C), and absolute risk of hypoglycaemia in the preceding month for the whole population (Panel D) based on mixed model analyses using restricted cubic splines in the subgroup of patients on stable non-injectable antidiabetic treatment

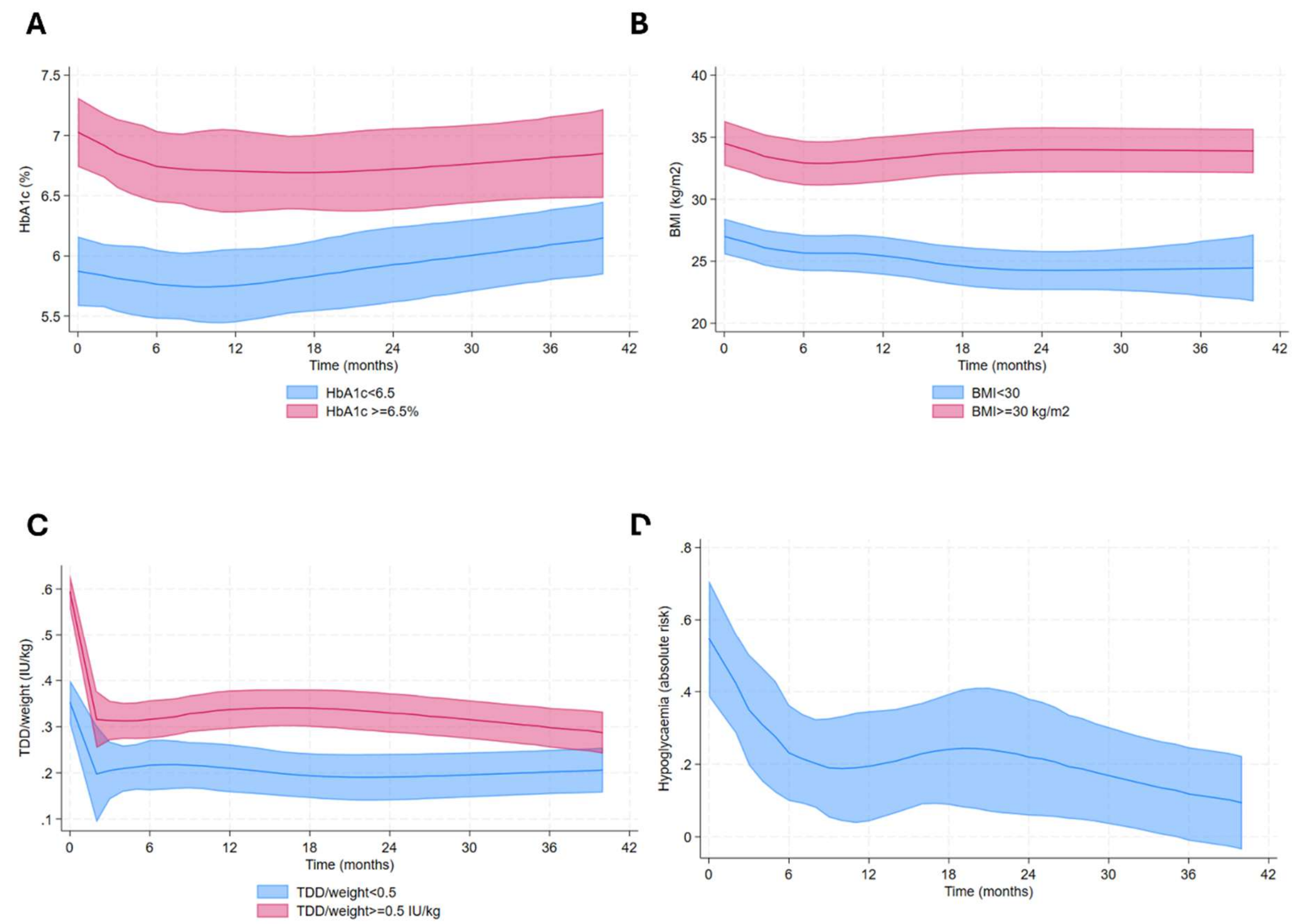

To convert HbA1c (%) to mmol/mol, the conversion formula is: HbA1c (mmol/mol)=10.9xHbA1c(%)-23.5

Supplementary Figure S2 – Estimated marginal means with 95% confidence bands of BMI by baseline weight normalized insulin dose among participants with higher (≥30 kg/m^2 – Panel A) and lower BMI (<30 kgm^2– Panel B) at baseline based on mixed model analyses using restricted cubic splines in the subgroup of patients on stable non-injectable antidiabetic treatment

A

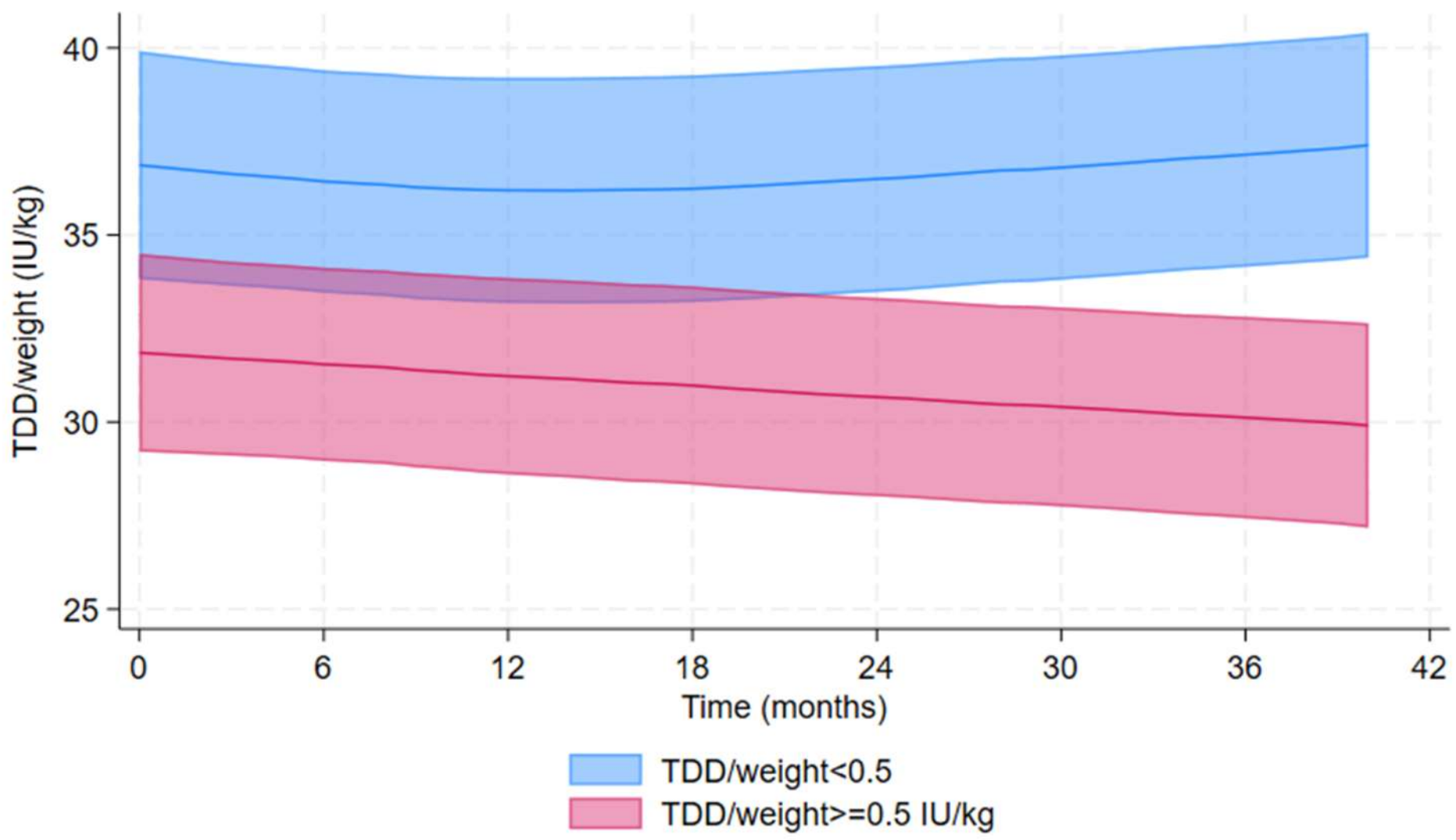

B

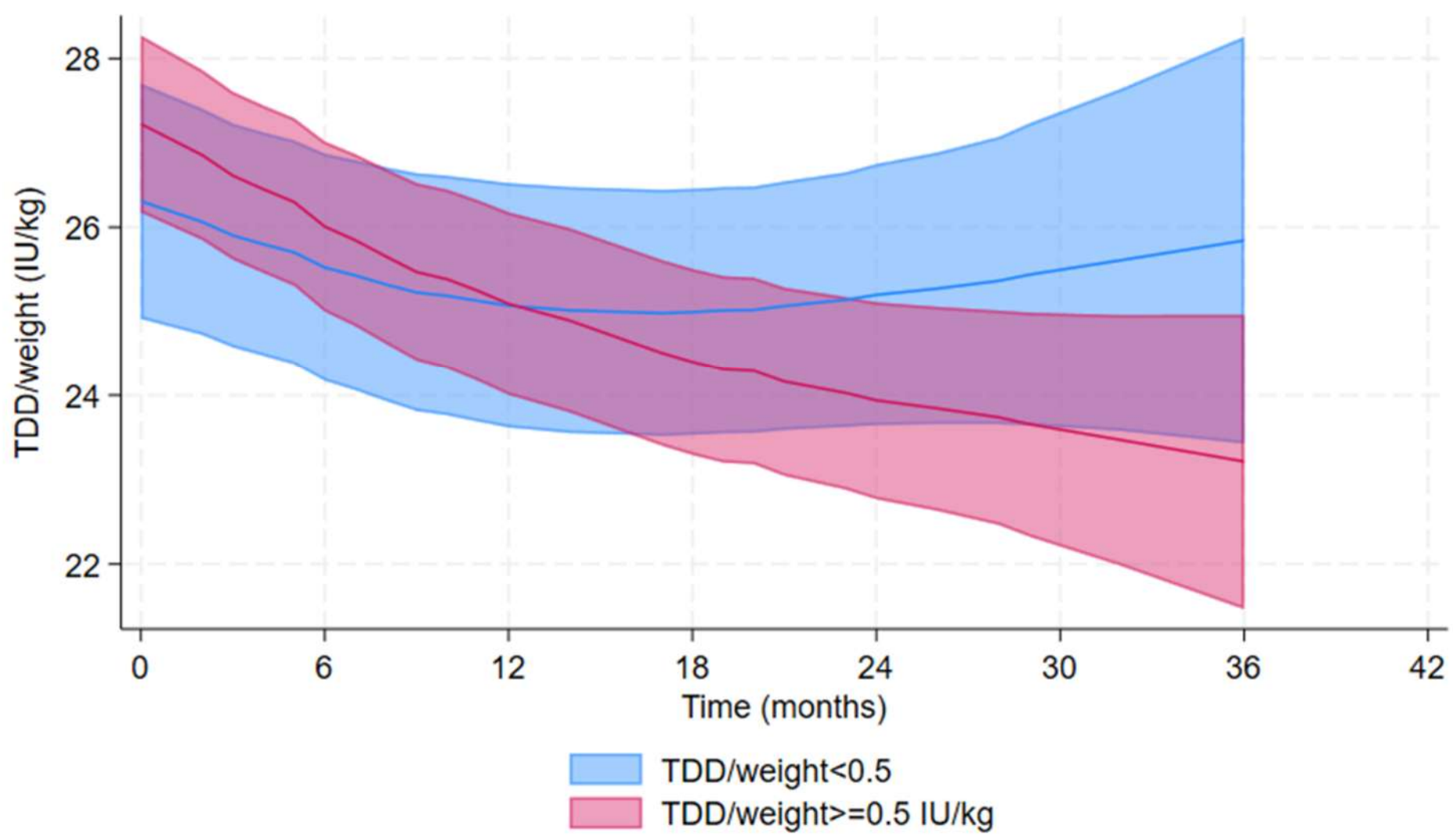

Supplement: Supplementary file 1 [file DataSheet1.pdf]
